# Shape-selected nanocrystals for in situ spectro-electrochemistry studies on structurally well defined surfaces under controlled electrolyte transport: A combined in situ ATR-FTIR/online DEMS investigation of CO electrooxidation on Pt

**DOI:** 10.3762/bjnano.5.86

**Published:** 2014-05-28

**Authors:** Sylvain Brimaud, Zenonas Jusys, R Jürgen Behm

**Affiliations:** 1 Institut für Oberflächenchemie und Katalyse, Ulm University, Albert-Einstein-Allee 47, D-89081 Ulm, Germany

**Keywords:** CO oxidation, electrocatalysis, in situ spectro-electrochemistry, Pt, shape selected nanocrystals

## Abstract

The suitability and potential of shape selected nanocrystals for in situ spectro-electrochemical and in particular spectro-electrocatalytic studies on structurally well defined electrodes under enforced and controlled electrolyte mass transport will be demonstrated, using Pt nanocrystals prepared by colloidal synthesis procedures and a flow cell set-up allowing simultaneous measurements of the Faradaic current, FTIR spectroscopy of adsorbed reaction intermediates and side products in an attenuated total reflection configuration (ATR-FTIRS) and differential electrochemical mass spectrometry (DEMS) measurements of volatile reaction products. Batches of shape-selected Pt nanocrystals with different shapes and hence different surface structures were prepared and structurally characterized by transmission electron microscopy (TEM) and electrochemical methods. The potential for in situ spectro-electrocatalytic studies is illustrated for CO_ad_ oxidation on Pt nanocrystal surfaces, where we could separate contributions from two processes occurring simultaneously, oxidative CO_ad_ removal and re-adsorption of (bi)sulfate anions, and reveal a distinct structure sensitivity in these processes and also in the structural implications of (bi)sulfate re-adsorption on the CO adlayer.

## Introduction

Since the pioneering work of Bewick and coworkers [1–2], in situ infrared (IR) spectro-electrochemistry has been widely used to probe adsorbed species at the electrified solid/liquid interface under potential control. Among others, IR spectro-electrochemistry [3] was successfully employed to investigate the relations between electrode surface structure and the binding modes of adsorbed species [4–10], to identify poisoning species and adsorbed intermediates formed during electrocatalytic reactions [2,11–13], and this way to unravel mechanistic details, or to resolve the structure of the water adlayer [14]. A major drawback of this method, at least for certain applications, is, however, that so far it is not possible to study electrochemical/electrocatalytic processes under enforced and well defined mass transport conditions on structurally well defined samples such as single crystalline electrodes. In the commonly applied external reflection configuration, where the working electrode is pressed against an IR-transparent window in order to minimize IR absorption by water in the aqueous electrolyte, it is possible to perform in situ spectroscopy (infrared reflection–absorption spectroscopy, IR-RAS) on single crystal surfaces, but the thin electrolyte layer precludes any significant mass transport [4–6]. On the other hand, mass transport of the electro-active species can be enforced and properly controlled employing the so-called internal reflection configuration (attenuated total reflection Fourier transform infrared spectroscopy, ATR-FTIRS) [12,15]. In this case, however, the working electrode consists of a structurally little defined polycrystalline metal film, which is directly deposited on the flat side of an IR-transparent reflecting element, as had been demonstrated, e.g., for Pt [12,16–17] or Au [18–19] films. We had later shown that in this geometry the reaction cell can be coupled in addition with a DEMS (differential electrochemical mass spectrometry) setup, allowing electrochemical measurements, IR spectroscopic detection of adsorbed species such as reaction intermediates or reaction side-products, and mass spectrometric detection of volatile reaction (side) products at the same time [15,20].

For spectro-electrochemical studies of reactions sensitive to mass transport effects, which includes many electrocatalytic reactions, it would be highly desirable to devise a way that allows us to use structurally better defined samples in an ATR configuration, e.g., to elucidate structural effects in the reaction. One possible approach would be use preferentially shaped nanoparticles of the respective material with well defined facet orientations (shape-selected nanocrystals), which are deposited on a chemically inert, IR transparent and electrically conducting film covering the ATR prism. Following these lines we have recently shown that ca. 10 nm (111)-Pt nano-octahedrons deposited on an Au film substrate, where the latter acts a chemically inert, but electrically conducting substrate, can be employed for in situ ATR-FTIRS investigations [21–22]. In that work we could provide first evidence that these working electrodes present voltammetric features and vibrational properties of adsorbed species which differ drastically from those of a polycrystalline Pt film and which are characteristic for a Pt(111) surface with a well ordered surface. In the present paper we will further explore the potential of this approach, extending this to nanoparticles with different shapes/facet orientations and to a more detailed characterization of the structural and electrochemical/electrocatalytic properties of these electrodes. Employing different types of shape-selected Pt nanocrystals (ca. 10 nm cubes, octahedrons and truncated octahedrons), we will quantitatively evaluate the contributions of different low index facet orientations to the respective properties, comparing results from electron microscopy, electrochemical measurements, in situ IR spectroscopic and mass spectrometric measurements. In the end, this aims at the possibility to conduct spectro-electrochemical studies under enforced and well controlled mass transport conditions on electrodes with well defined structural properties tailored for specific applications.

In the following, we will after a brief description of the experimental procedures and set-up, including the nanoparticle preparation, first describe the results of the structural characterization of 3 batches of differently shaped Pt nanoparticles by electron microscopy, and electrochemical techniques, including H-underpotential deposition as well Ge and Bi deposition (section ‘Characterization of the Pt samples’). Subsequently, we will characterize the CO adsorption properties (section ‘ATR-FTIRS characterization of structurally well defined Pt nanocrystals’) and the re-adsorption of (bi)sulfate anions upon oxidative removal of CO_ad_ (CO_ad_ stripping) (section ‘Monitoring sulfate re-adsorption during CO_ad_ oxidation’) by combined in situ ATR-FTIRS and DEMS. The latter allows us to disentangle the contributions from the two processes, CO_ad_ oxidation and (bi)sulfate re-adsorption, to the Faradaic current for the different surface orientations, illustrating the potential of this approach.

## Results and Discussion

### Characterization of the Pt samples

**Figure 1:**
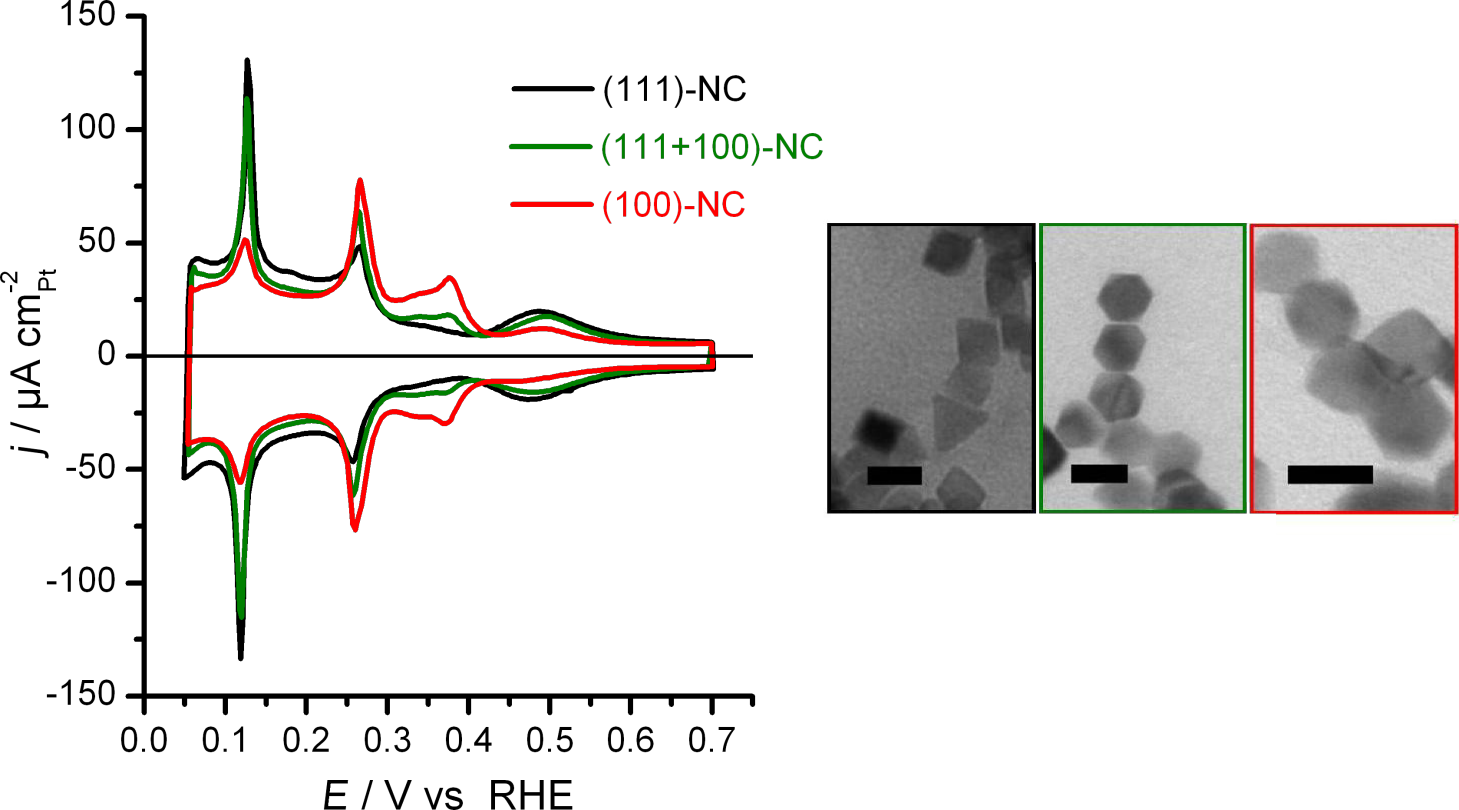
Left: Hydrogen-region voltammograms recorded at 50 mV s^−1^ in 0.5 M H_2_SO_4_. Black, green and red traces correspond to (111)-NC, (111+100)-NC and (100)-NC samples, respectively. Right: Representative BFTEM images for (111)-NC, (111+100)-NC and (100)-NC, respectively from left to right. The scale bar indicates 10 nm.

For the present investigations we used three different batches of shape-selected Pt nanocrystals named after their dominant surface orientation. As illustrated in Figure 1, the hydrogen region voltammograms display current features which are characteristic of well ordered low index Pt surfaces [23–24]. Sharp and symmetric hydrogen sorption peaks are convincing indicators for both a well ordered Pt nanocrystal surface structure and the cleanness of the experimental setup and procedures. The hydrogen-region voltammograms obtained for such samples are approximated as weighted sum of the respective features of extended low Miller index single crystal electrode contributions. For more details we refer to [24–25] and references therein. Briefly, and focusing on the most significant current features, the (111)-NC sample contains 70% of ca. 10 nm octahedrons and exhibits the most pronounced broad peak at ca. 0.50 V, which is associated to (bi)sulfate adsorption on (111) oriented facets. On the other hand, the two peaks between 0.30 and 0.40 V vs RHE, which are characteristics of (100) oriented surfaces [26], are absent for the (111)-NC sample and particularly well-developed for the (100)-NC sample, where the latter one contains a significant amount of nanocubes with ca. 10 nm size. An “intermediate” (111+100)-NC sample containing essentially ca. 10 nm cuboctahedrons has also been prepared, its hydrogen region voltammogram displays current features which are a combination of both types described before. The relative amounts of the respective orientations obtained from a qualitative analysis of the *H*_upd_ peaks are in good agreement with the results of the analysis of the bright field transmission electron microscope (BFTEM) images (see Table 1).

**Table 1:** Shape statistics from BFTEM analysis of the projected areas.

sample	2D projected area				
	regular hexagon	irregular hexagon	rhombus	square	others

(111)-NC	0%	12%	70%	0%	18%
(111+100)-NC	48%	18%	23%	0%	11%
(100)-NC	25%	31%	7%	22%	15%
plausible 3D body	cuboctahedron	truncated octahedron or cuboctahedron	octahedron	cube	others or undefined

Results from the more precise quantification by Ge and Bi adatom deposition, which specifically probe the contributions from ordered (100) and (111) surface orientations, respectively, are summarized in Table 2. They agree well with results from hydrogen region voltammograms and BFTEM. It is important to realize that for all three samples the amount of well ordered facet area determined this way accounts for about 50% of the electrochemically active surface area, as determined by *H*_upd_, with different ratios between (111) and (100) surface orientation depending on the nanocrystal shapes.

**Table 2:** Fraction of ordered facets relative to the total surface area of the Pt nanocrystals, as determined by Bi and Ge adatom deposition, charge displaced (*q*_dis_) during adsorption of CO at *E*_ads_ = 0.1 V, estimate of the potential of zero total charge (*E*_pztc_), and the CO_ad_ saturation coverage (θ_CO_) for each sample. In brackets are the uncertainties of the measurement as derived from repeated measurements.

sample name	fraction of (100) facets / %	fraction of (111) facets / %	*q*_dis_ / µC cm^−2^	*E*_pztc_ / V	measured θ_CO_

(111)-NC	6	49	148 (±4)	0.259 (±0.004)	0.66 (±0.03)
(111+100)-NC	17	32	150 (±1)	0.280 (±0.002)	0.69 (±0.03)
(100)-NC	37	16	146 (±3)	0.304 (±0.005)	0.72 (±0.03)

The charge displaced during CO adsorption at 0.10 V is about 150 µC cm^−2^ for all three samples investigated, independently of the Pt nanocrystal surface structure (see Table 2). This allows us to determine the *E*_pztc_ [27–29] for each of the working electrodes prepared from one of the three different batches of nanocrystals (see section ‘Preparation and characterization of the Pt nanocrystals’). The resulting values for the *E*_pztc_ are 0.259 (±0.004) V, 0.280 (±0.002) V and 0.304 (±0.005) V for the (111)-NC, (111+100)-NC and (100)-NC samples, respectively (see also Table 2). The average charge for a polycrystalline electrode can be considered as a weighted sum of the contribution from each low-index facet orientation, weighted by their respective abundance [30–31]. This has to consider, however, also, contributions from the areas with non-perfect surface structure, including steps, edges etc., according to the Bi and Ge probing experiments contribute around 50% to the active surface area of each of the different batches of nanocrystals (see Table 2). Including this, the measured values agree well with the trend expected from the *E*_pztc_ values of the low-index Pt single crystal electrodes (0.15, 0.38 and 0.32 V for Pt(110), Pt(100) and Pt(111) in 0.5 M H_2_SO_4_, respectively [27]. Similar to the case of extended single crystal electrodes, *E*_pztc_ increases with the relative fraction of (100) facets for the nanocrystals investigated here (see Table 2).

In total, following the procedure developed by the Feliu group for the characterization of shape-selected Pt nanocrystals [24–25,29], we have three structurally well-defined and well-characterized samples of Pt nanocrystals available for the in situ IR investigations.

### ATR-FTIRS characterization of structurally well defined Pt nanocrystals

**Figure 2:**
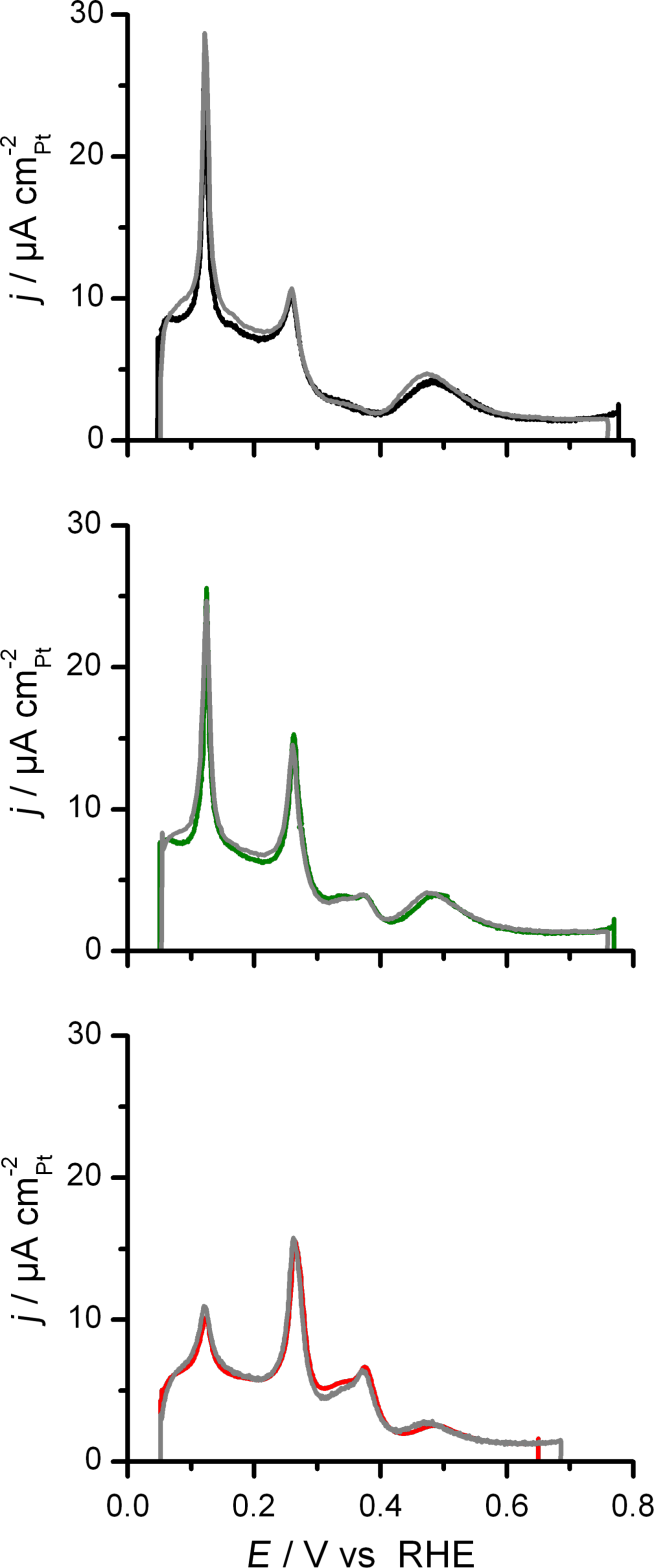
Comparison of the anodic scan of the hydrogen-region voltammograms recorded in the beaker cell (colored lines) and in the spectro-electrochemical flow cell (grey lines) at 10 mV s^−1^ in 0.5 M H_2_SO_4_. From top to bottom: (111)-NC, (111+100)-NC and (100)-NC.

Next, the adsorption properties of the structurally well defined Pt nanocrystals are characterized by FTIR spectroscopy, using adsorbed CO as probe molecule. For these measurements, the Pt nanocrystals are supported on a polycrystalline Au film pre-deposited on the flat side of a hemispherical Si prism for in situ ATR-IR investigations (see section ‘Preparation and characterization of the Pt nanocrystals’). Before starting with the FTIR measurements, it is important to ensure that the experimental conditions, in particular the cleanness of sample and setup, resemble those in the beaker cell measurements. Figure 2 displays a comparison of the anodic part of the hydrogen-region voltammograms recorded in the beaker cell and in the thin-layer spectro-electrochemical flow cell for the three samples investigated. Obviously, they are identical. For investigations of the IR vibrational properties of adsorbed CO, gold films have the advantage that CO is adsorbed only in dynamic equilibrium with dissolved CO in the electrolyte [21,32]. Thus, after complete removal of CO from solution by purging of the flow cell with CO-free electrolyte, only the irreversibly adsorbed CO on the Pt nanocrystal surface remains.

**Figure 3:**
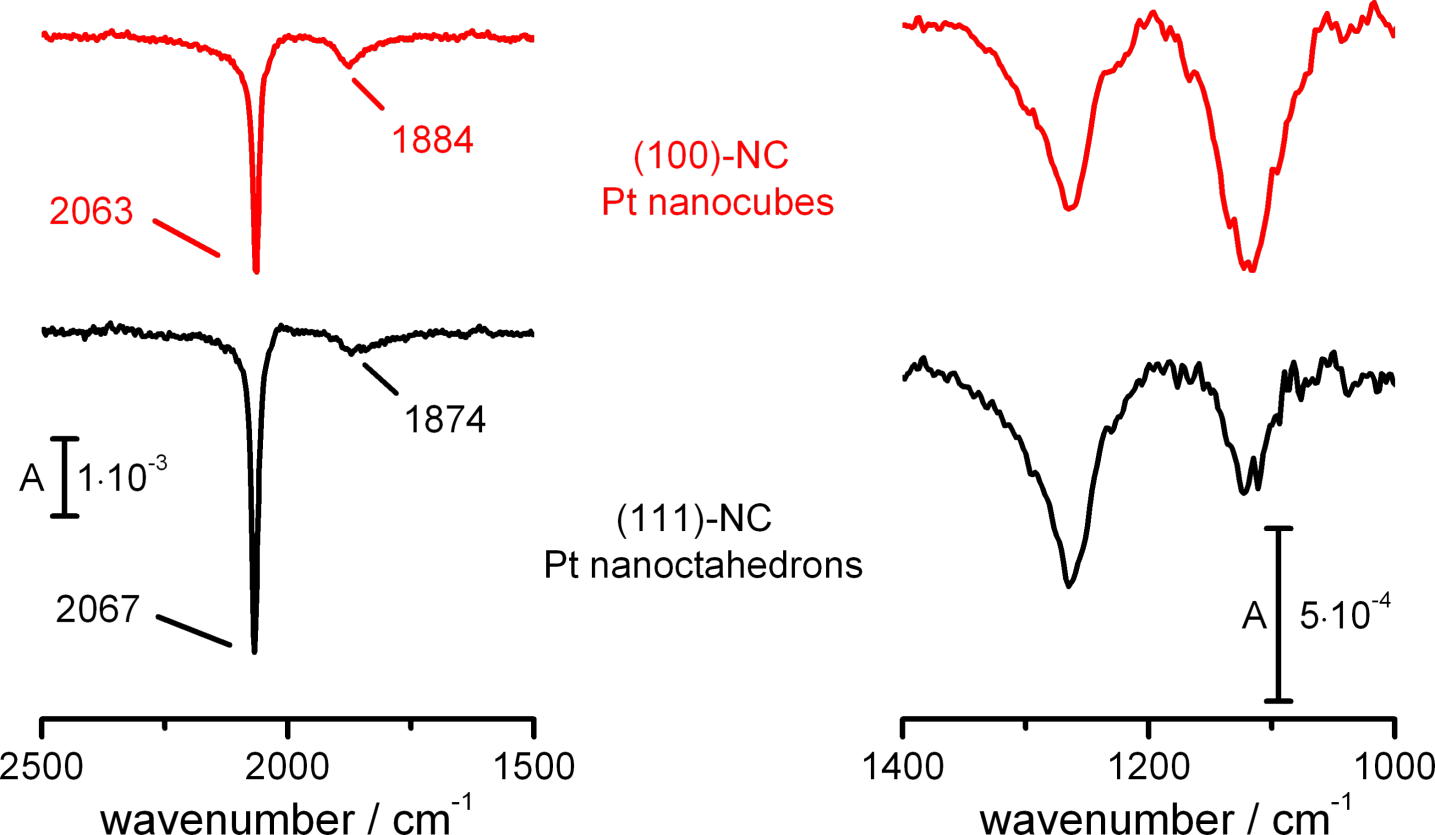
IR vibrational spectra of irreversibly adsorbed CO recorded at 0.10 V (left panel) and of adsorbed (bi)sulfate recorded at 0.75 V (right panel) on (100)-NC (top) and (111)-NC (bottom). *R*_0_ was recorded at the same potential in the absence of CO (left panel). All experiments were conducted in 0.5 M H_2_SO_4_ and all spectra were acquired in the absence of dissolved CO.

Figure 3 compares ATR-FTIR spectra in the spectral regions for adsorbed CO and adsorbed (bi)sulfate anions recorded on the (111)-NC and (100)-NC samples. The inversion of the band polarity (abnormal IR effect) apparent from these spectra is well known for nanoparticle samples and has already been discussed elsewhere [21,33]. This does not, however, limit the interpretation of these spectra. After CO adsorption at 0.10 V and complete removal of dissolved CO, which was achieved by an electrolyte exchange under continuous potential control [12], two IR bands are observed in the 1500–2500 cm^−1^ region of the spectrum, which are due to linearly (CO_L_, higher wave number) and bridge-bonded (CO_B_, lower wave number) adsorbed CO, respectively [3,7–8,34]. For (111)-NC, the band corresponding to CO_L_ is blue-shifted by 4 cm^−1^ compared to that on (100)-NC while, oppositely, the band corresponding to CO_B_ is red-shifted by 10 cm^−1^. The lower wave number obtained for CO_L_ adsorbed on the (100)-NC than on the (111)-NC sample agrees well with trends expected from CO adsorption on Pt(100) and Pt(111) surfaces [8,35–36]. Furthermore, we find a slightly smaller difference in wave numbers (Δ
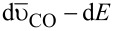
) between the two binding modes of CO_ad_ for (100)-NC than for (111)-NC. Also, the relative fraction of bridge-bonded CO_ad_ seems to be higher for the former nanocrystal sample than for the latter one. However, since the IR band intensity is known to not directly reflect the CO_ad_ coverage/population (bonding mode) [37], in particular not at high coverages, we will refrain from a more quantitative discussion. In total, the IR observations are fully consistent with in situ IR observations on CO_ad_ saturated low Miller index single crystal electrodes [8,35–36].

The IR spectral region characteristic of adsorbed (bi)sulfate (1050–1350 cm^−1^ [38–39]) is displayed in the right panel of Figure 3. Two bands are observed at ca. 1260 cm^−1^ and 1120 cm^−1^, with different relative intensities depending on the surface structure of the respective nanocrystals. Inspection of previously published FTIR spectra obtained in an external reflection configuration on extended Pt single crystal electrodes [9–10] reveals that the band at higher wave number is characteristic for adsorption on Pt(111), while that at lower wave number is favoured for Pt(100). The different intensities in the two peaks reflect the influence of the different facet orientations. Thus, also in this case the IR data on shaped-selected nanocrystals are fully consistent with observations on extended single crystal electrodes.

Overall, the IR vibrational properties of adsorbed species (CO_ad_, adsorbed (bi)sulfate) on shape-selected nanocrystals confirm the findings from electron microscopy and electrochemical measurements that the use of shape selected nanoparticles results in structurally well defined electrodes, with dominant structures equal to those of the respective single crystal electrodes. The absorbance by the underlying Au film is sufficiently low for in situ ATR-IR measurements, opening a door for IR measurements on structurally well defined electrodes with tunable surface structure under enforced and controlled electrolyte mass transport.

### Monitoring sulfate re-adsorption during CO_ad_ oxidation

**Figure 4:**
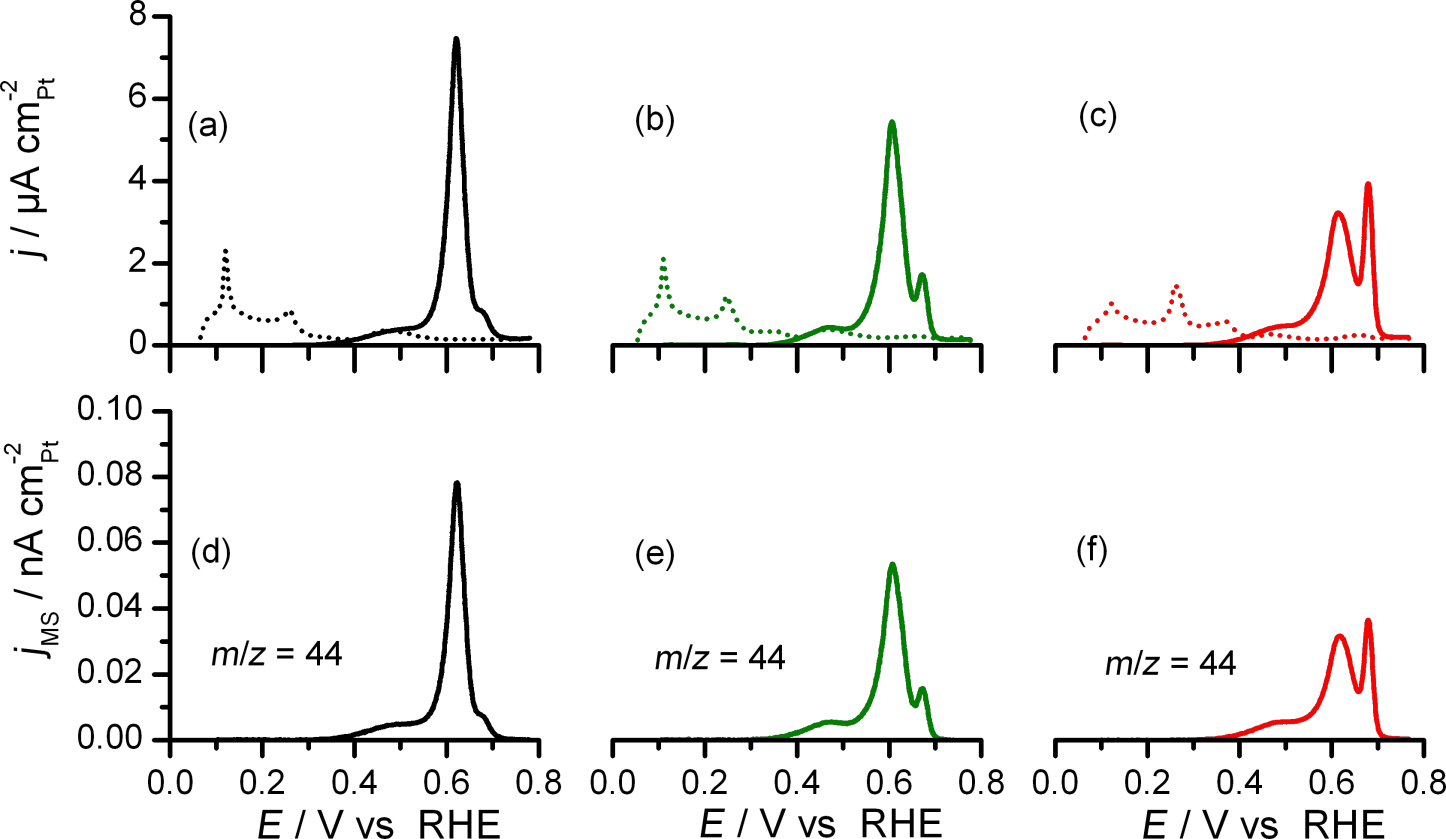
First (solid lines) and second (dashed lines) positive going scans of CO stripping voltammograms (a–c) and mass spectrometric current of the *m*/*z* = 44 signal (CO_2_ detection) (d–f) for (111)-NC (a,d), (111+100)-NC (b,e) and (100)-NC (c,f). H_2_SO_4_ 0.5 M, *v* = 1 mV s^−1^.

As a first application of this newly developed tool for in situ spectro-electrochemical studies we investigated the potentiodynamic oxidation of a pre-adsorbed CO adlayer (CO_ad_ stripping). Combination of the thin-layer spectro-electrochemical flow cell with the DEMS setup allows for an online detection of the CO_2_ produced upon CO_ad_ electro-oxidation. Results from potentiodynamic oxidative removal of CO_ad_, which was pre-adsorbed at 0.1 V, are shown in Figure 4. The Faradaic currents recorded during the first and subsequent anodic scans are displayed in Figure 4a–c for the three samples investigated, the corresponding mass spectrometric (MS) currents (*m*/*z* = 44) resulting from detection of CO_2_ are displayed in Figure 4d–f. For all samples, CO_ad_ electro-oxidation starts at 0.30 V, followed by the well-known pre-peak for CO_ad_ oxidation, and then by two oxidation peaks, whose maxima are located at ca. 0.61 and 0.67 V, respectively. The Faradaic current profiles for the CO_ad_ stripping agree well with those obtained in beaker cell measurements on similar nanocrystals [40–41]. Also the potentials at the maxima of the oxidation peaks measured during potentiodynamic oxidation at 1 mV s^−1^ are in good agreement data obtained at faster scan rates and d*E*_peak_/d(log *v*) slopes reported previously [41]. The intensity of the second oxidation peak was found to be correlated with the fraction of (100) facets of the respective nanocrystal samples [41].

**Figure 5:**
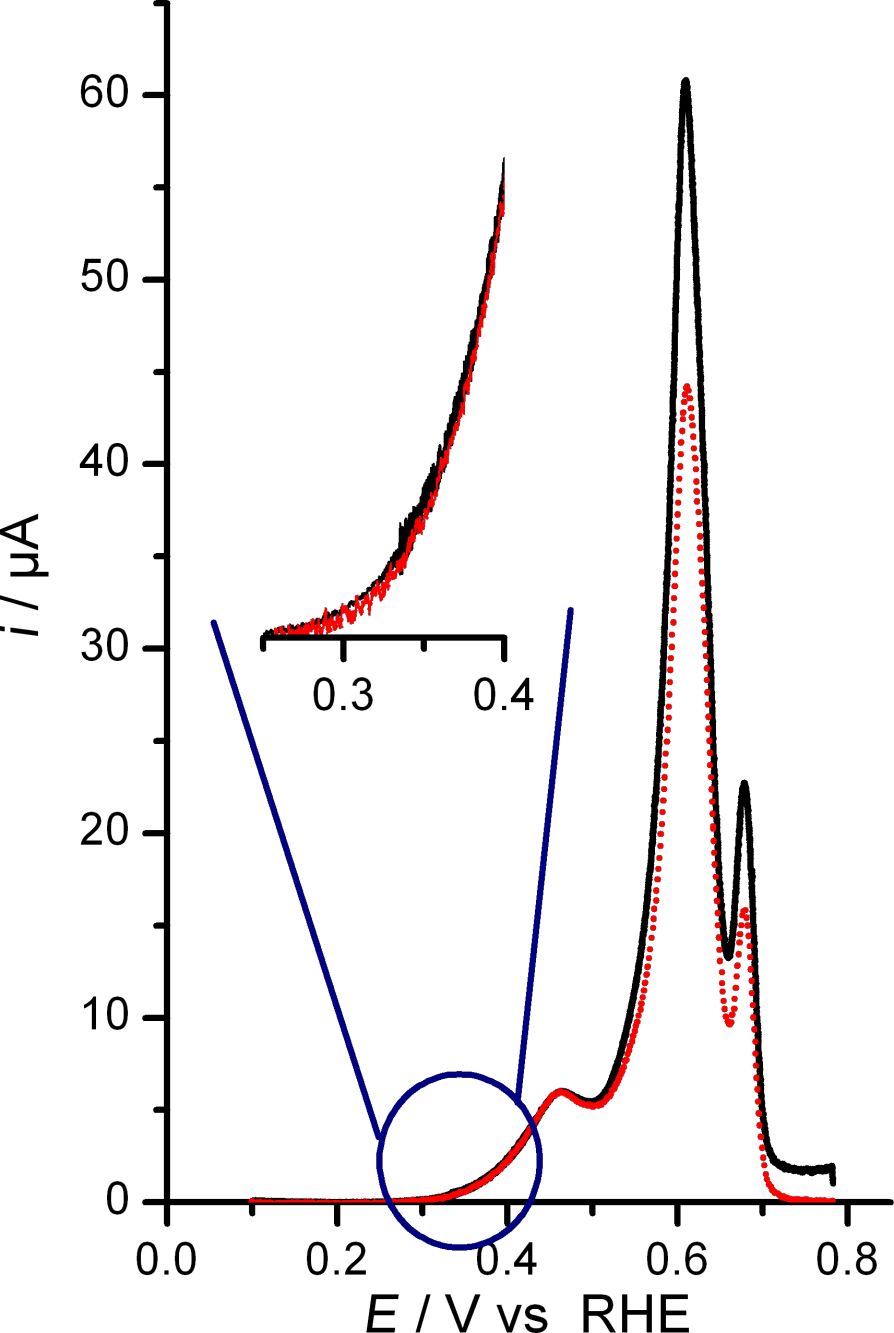
Normalized mass spectrometric current (*m*/*z* = 44, CO_2_ detection, red dotted line) and Faradaic current (black solid line) acquired during the CO_ad_ stripping experiment on (111+100)-NC (*v* = 1 mV s^−1^). The CO adlayer was formed by adsorption from CO saturated electrolyte at 0.10 V (0.5 M H_2_SO_4_).

The MS current related to CO_2_ detection can be transformed into the related Faradaic current from CO_ad_ oxidation via the collection efficiency K*. This is defined as the ratio between the MS current signal and the Faradaic current related to the respective process, multiplied by the number of electrons exchanged per product molecule in the reaction (in our case, oxidation of adsorbed CO to CO_2_ leads to 2 electrons exchanged per CO_2_ molecule) [20]. While in most cases K* is determined by comparing the charge under the oxidation peak corrected by a contribution of 20% from anion readsorption [42], we here followed a different route. In the present study, we determined the K* value by comparison of the Faradaic and mass spectrometric (*m*/*z* = 44) currents in the potential region 0.30–0.35 V, assuming that in this potential range, at the onset of CO_ad_ oxidation, Faradaic processes other than CO_ad_ electro-oxidation can be neglected, while at higher potentials other processes such as re-adsorption of anions or surface oxidation may take place as well [43–45]. An example of such kind of refined K* determination is shown Figure 5. In the pre-peak region (up to ca. 0.50 V), the perfect match between the Faradaic current for CO_2_ production (determined using the K* value determined in the range from 0.30 to 0.35 V) and the measured Faradaic current for CO_ad_ oxidation supports the hypothesis that CO_ad_ electro-oxidation is the only Faradaic process occurring in this potential region. Thus, in the pre-peak region, the small fraction of CO_ad_ removed (see below) does not result in sufficiently opened Pt-CO adlayer allowing for (bi)sulfate anions readsorption [45]. The K* values obtained in this potential range were in the range between 2 and 3 × 10^−5^ for all experiments, which is in good agreement with previous findings on our experimental setup [20]. With further increase of the electrode potential, the normalized MS current from CO_2_ detection deviates from the Faradaic current for CO_ad_ electro-oxidation. This indicates that another Faradaic process occurs, while CO_ad_ is removed by potentiodynamic oxidation. This difference is mainly attributed to (bi)sulfate anion re-adsorption and progressive double layer charging, which occurs while CO_ad_ is removed by potentiodynamic oxidation. Note that this direct procedure for determining the contribution of anion re-adsorption leads to results for the charge contribution from (bi)sulfate re-adsorption which are comparable with those of earlier (and more indirect) coulometric analysis (see below) [45–46]. Correspondingly, the difference between the normalized (mass spectrometric) current from CO_2_ detection and the Faradaic current from CO_ad_ electro-oxidation reflects the current associated with (bi)sulfate anion re-adsorption and double layer charging, while CO_ad_ is increasingly removed from the surface.

**Figure 6:**
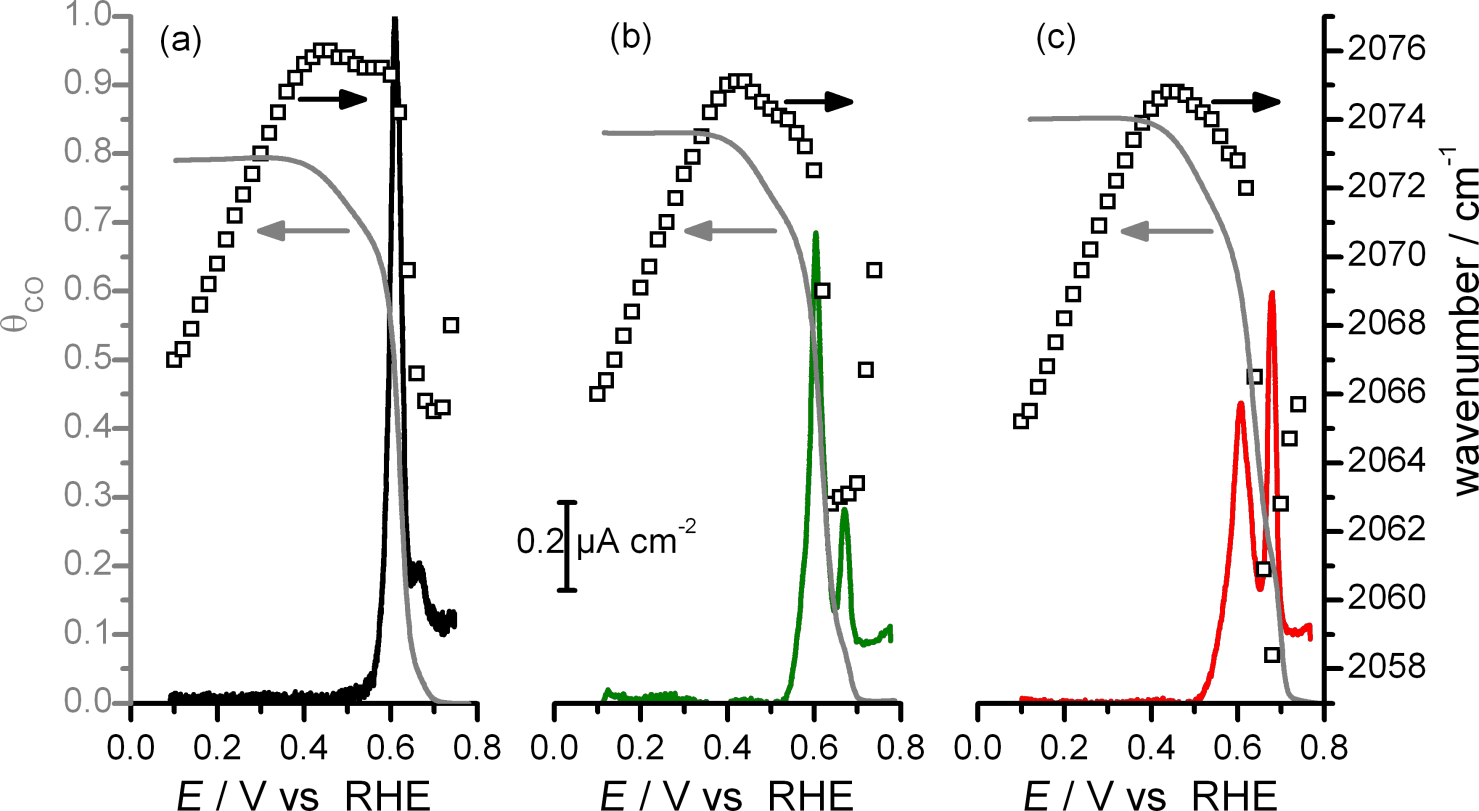
Plots of the currents from (bi)sulfate anions re-adsorption (colored traces), absolute CO_ad_ coverages (θ_CO_, grey traces) and IR band frequencies for linearly adsorbed CO (open square) as function of the electrode potential for (111)-NC (a), (111+100)-NC (b) and (100)-NC (c) (0.5 M H_2_SO_4_, *v* = 1 mV s^−1^).

Plots of the current associated to (bi)sulfate anions re-adsorption (and capacitive double layer charging) during potentiodynamic CO_ad_ oxidative removal are displayed in Figure 6 for the three samples of shape-selected nanoparticles investigated. To the best of our knowledge, this is the first time that these currents have been measured directly.

The charge which is not related to CO_ad_ electro-oxidation in CO_ad_ stripping voltammogram is estimated to ca. 19% of the total charge, in good agreement with the value obtained from a pure coulometric analysis developed on Pt single crystalline electrodes (≈20%) [45]. In the latter method, the charge correction is determined via the so-called “double-layer” correction, where the sum of the charge displaced upon CO adsorption at the onset of hydrogen adsorption and of the charge in a base CV between that potential and the upper limit of the CO_ad_ stripping peak are subtracted from the total charge measured for the electrooxidation of a saturated CO adlayer. The absolute CO_ad_ coverage (θ_CO_ , with θ_CO_ = 1 for 1 CO_ad_ per Pt surface atom) resulting from the formation of a CO adlayer at 0.10 V can now be estimated from the ratio between the charge related to CO_ad_ electro-oxidation and double the charge related to H adsorption, and assuming a hydrogen saturation coverage of 0.77 at 0.05 V [47], independent of the Pt surface structure. The charge related to H adsorption can be determined from the Faradaic charge under the anodic part of the hydrogen region voltammogram, corrected for the charge associated to anion sorption (obtained from Figure 6) and the double layer capacitive contribution. The resulting saturation coverages of the CO adlayer formed at 0.10 V are tabulated in Table 2 and range between 0.6 and 0.7 CO_ad_ per Pt atom, in good agreement with findings for extended Pt single crystal surfaces/electrodes [45]. To illustrate the different potential dependences in CO_ad_ removal, we also plotted the evolution of the absolute CO_ad_ coverage as a function of the electrode potential during CO_ad_ stripping for the three nanocrystal samples (see Figure 6). Just as an example, at 0.64 V, 90%, 80% and 60% of the CO_ad_ has been removed from the nanocrystal surface for the samples (111)-NC, (111+100)-NC and (100)-NC, respectively.

The changes in the CO adsorption properties were monitored by following the frequency shifts of the IR band associated to linearly adsorbed CO, which are also plotted in Figure 6. They follow classical descriptions reported previously [5,34,43,48]. First, between 0.10 and 0.45 V, the CO_L_ band is linearly blue-shifted due to the effect of the applied electric field, i.e., reflecting the so-called Stark effect. The slope of between 30 and 33 cm^−1^ V^−1^ agrees well with previous findings on Pt surfaces [15,49–50]. Then, as the CO_ad_ coverage decreases, the CO_L_ band is dramatically red-shifted. This observation is generally interpreted as due to a stronger metal–CO_ad_ binding, caused by a lowering of the CO_ad_–CO_ad_ lateral repulsion [5,34,48]. Finally, at potentials higher than 0.65 V, this changes again into a blue shift of the CO_L_ band, leading to a “U-shape” of the 
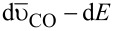
 plot. It should be noted that, as shown Figure 6, the extent of amplitude of the “U-shape” depends on the structure of the nanocrystal surface, it is more pronounced as the fraction of (100) facets increases. This blue shift had been attributed to a compression of the remaining CO due to anion re-adsorption [43–44,51]. The plot of the current associated to anion re-adsorption displayed in Figure 6 supports this hypothesis. In fact, the intensity of the second peak of (bi)sulfate re-adsorption and the extent of the blue-shift at high potentials seem to be correlated, with both of them increasing as the fraction of ordered (100) area on the nanocrystal surface increases. The correlation between (bi)sulfate re-adsorption and CO_ad_ compression clearly illustrates the role played by anions for CO_ad_ bonding and for CO_ad_ electro-oxidation. Finally, the difference in anion re-adsorption for the three different samples illustrates also the structure sensitivity of this process.

## Conclusion

Employing in situ ATR-FTIR spectroscopy and mass spectrometry in a combined ATR-FTIR/DEMS flow cell set-up, we could demonstrate that shape-selected and structurally well characterized nanocrystals deposited on a Au film/Si substrate offer an attractive opportunity for performing spectro-electrochemical and in particular spectro–electrocatalytic measurements under enforced and well controlled electrolyte mass transport conditions on structurally well defined electrode surfaces. The surface morphology and structure of the shape-selected nanocrystals, which are prepared by colloidal synthesis, can be varied in a controlled way over a wide range. TEM, electrochemical and in situ ATR-FTIR characterization agreeingly demonstrated that the surface of the nanocrystals consists predominantly of well ordered facets with low index orientation, whose relative contribution depends on the synthesis conditions. Comparison with extended single crystal electrodes revealed similar electrochemical and adsorption/reaction characteristics for low index facets and the corresponding extended single crystals, as evidenced by both voltammetric traces and the vibrational properties of adsorbed CO.

The possibility to prepare tailored model electrodes, whose active surface is dominated by one or more specific structural elements, offers new perspectives for spectro-electrochemical investigations of the relationship between reaction mechanism/kinetics, electrolyte mass transport and electrode surface structure. The potential of such kind of measurements is illustrated for potentiodynamic CO_ad_ oxidation, where the simultaneous online DEMS detection of the CO_2_ produced during the oxidative removal of CO_ad_ and the measurement of the overall Faradaic current allow us to directly measure and separate the contribution from re-adsorption of (bi)sulfate anions to the Faradaic current. Finally, in situ ATR-FTIRS measurements performed in parallel reveal changes in the vibrational properties which can be traced back to the electrostatic Stark effect, changes in the CO_ad_ coverage and hence in the structure of the CO adlayer with increasing CO_ad_ removal, and finally similar changes induced by increasing re-adsorption of (bi)sulfate anions. Due to the strict time correlation between the different measurements the contributions from the different processes can be disentangled. Overall, this yields a detailed picture of the different processes occurring during the CO_ad_ oxidation reaction, on a molecular scale and specific for selected surface structures, which will be important for bulk reaction studies, e.g., of fuel cell relevant reaction, which are planned for the future.

While this was demonstrated for Pt nanocrystals, other metals are equally accessible, leaving this as a generally applicable approach for in situ spectro-electrochemical studies on structurally well defined electrodes under enforced and controlled electrolyte mass transport conditions.

## Experimental

### Preparation and characterization of the Pt nanocrystals

Batches of shape-selected Pt nanocrystals of different shapes were prepared by a colloidal synthesis method mainly developed by the El-Sayed group [52–54]. This synthesis method leads to ca. 10 nm sized nanocrystals with well ordered facets in low index orientations, whose voltammetric traces resemble those of flame annealed extended low Miller index single crystalline electrodes, as demonstrated previously by the Feliu group [25,40,55]. Prior to the synthesis, the reaction vessel was first cleaned by aqua regia to remove metal traces which could act as nucleation center, then with saturated alkaline solution to remove organic contaminants which could influence the growth process, and finally boiled in ultrapure water (MilliQ^®^, 18 MΩ cm). Briefly, the synthesis method consists of the reduction of a Pt salt (Alfa Aesar) dissolved in deaerated ultrapure water by hydrogen bubbling (5.0, Westfalen) in the presence of sodium polyacrylate (NaPA, *M*_w_ 2100, Sigma-Aldrich). The relevant synthesis parameters are given Table 3. After 10 min of hydrogen bubbling, the reaction vessel was sealed and left over night, protected from the light. Then, the precipitation of the nanocrystals was initiated by adding a few NaOH pellets. Cleaning of the nanocrystals was obtained by successive precipitation/redispersion in ultrapure water. Finally, the resulting batches of nanocrystals were stored in a few millilitres of ultrapure water. All chemicals and gases were used as received without any further purification.

**Table 3:** Relevant synthesis parameters and Pt precursors.

sample name	Pt precursor	NaPA/Pt ratio	initial pH	aging of the Pt solutions

(111)-NC	H_2_PtCl_6_·6H_2_O	5	7	1 day
(111+100)-NC	K_2_PtCl_4_	1	8	3 days
(100)-NC	K_2_PtCl_4_	12	7	3 days

Sizes and shapes of the nanocrystals were characterized by bright field transmission electron microscopy (BFTEM) (Philips CM 200 kV). A droplet of an aqueous suspension of nanocrystals was deposited on a carbonized Cu grid (Plano) and dried slowly. Free software (ImageJ, National Institute of Health / USA) was used to analyse the images.

The surface structure of the different batches of nanocrystals was furthermore characterized by electrochemical methods. The measurements were conducted in a classical three electrode beaker cell. The nanocrystals were deposited on a pure gold hemispherical bead by pipetting a droplet of water containing nanocrystals and subsequent drying under a N_2_ flow. A qualitative picture of the Pt nanocrystals surface structure was obtained by potentiodynamic adsorption/desorption of hydrogen (in the so-called hydrogen region of the voltammogram). The surface areas of well ordered facets of specific orientation exhibited by the nanocrystal surface (total electrochemically active Pt surface area ca. 0.1 cm^2^), were determined by coulometric analysis of the redox processes on a surface covered by a Ge or Bi adlayer generated by spontaneous deposition, following a method developed by the Feliu group and detailed in [56–57]. For determining the area of (100) oriented facets, we used spontaneous deposition of a Ge adlayer, which was performed by dipping the working electrode 5 min into a 10^−2^ M GeO_2_ (Aldrich, 99.99% purity) containing 1 M NaOH solution. For determination of the (111) surface area, a similar process was employed using Bi deposition, where the working electrode was dipped for 5 min in saturated Bi_2_O_3_ (Aldrich, 99.99% purity) in 0.5 M H_2_SO_4_ solution. Subsequently, the modified working electrode was introduced into the electrochemical cell under potential control at 0.15 V. Then, the electrode was cycled a few times between 0.15 V and 0.60 V for the Ge-modified Pt electrodes and between 0.15 V and 0.75 V for the Bi-modified Pt electrodes, and the coulometric charge associated with the surface oxidation/reduction of the Bi/Ge adlayer was determined and correlated to the amount of (111)/(100) ordered domains [56–57].

The potential of zero total charge (*E*_pztc_) was determined from the potential where the total charge passed in the base CV (hydrogen region) is equal to the charge displaced during CO adsorption at 0.10 *V*_RHE_ (*q*_dis_). This provides a direct determination of the *E*_pztc_ [27–29].

### Electrochemical setup

All electrochemical measurements, both in the beaker cell and in the in situ IR spectro-electrochemistry were conducted in O_2_-free 0.5 M H_2_SO_4_ supporting electrolyte, and freshly prepared from ultrapure water (MilliQ^®^, 18 MΩ cm) and conc. H_2_SO_4_ (Suprapur, Merk). A home-made hydrogen reference electrode (RHE) served as reference electrode, and all potentials given in the paper are referenced versus RHE, a gold foil was used as counter-electrode. A Pine model AFRDE-5 analog potentiostat interfaced to a computer was used for potential control and data acquisition.

#### ATR-FTIR spectro-electrochemical measurements

The spectro-electrochemical setup and the dual thin-layer flow cell used in this study were described in detail in [12,20]. The in situ FTIRS measurements in an attenuated total reflection (ATR) configuration were carried out using a BioRad FTS 6000 spectrometer equipped with a HgCdTe (MCT) detector, cooled with liquid nitrogen. The spectral resolution was set to 4 cm^−1^. The absorption is given in absorbance units defined as A = −log(*R*/*R*_0_), where *R* and *R*_0_ denote the reflectance at a given potential and at the reference potential, respectively. The respective reference potentials for *R*_0_ are specified in the figure captions.

For the FTIRS measurements the Pt nanocrystals were deposited on a thin Au film serving as chemically inert and stable and electrically conducting substrate, which in turn is deposited on a Si prism. The Au films have to be thin enough to be FTIR transparent and thick enough to exhibit sufficient electric conductivity and fully cover the Si substrate. The gold thin film was prepared by electroless deposition on the flat plane of a Si prism, using the procedure published by Miyake et al. [18]. After polishing and cleaning of the Si prism, its flat surface was dipped into 40% NH_4_F (BASF, Selectipure grade) in order to remove the oxide layer and to obtain a H-terminated Si surface, which improves the adhesion of the film. The gold plating solution consisted of a 1:1:1 mixture of 2% HF (Merck, suprapure grade), 0.03 M NaAuCl_4_ (Alfa Aesar) and 0.3 M Na_2_SO_3_ + 0.1 M Na_2_S_2_O_3_ + 0.1 M NH_4_Cl (all from Merck, pro analysi grade). This freshly prepared solution was pipetted onto the Si–H surface at 50 °C. After 80 s, the resulting film was rinsed with ultrapure water and dried under a N_2_ stream. After pipetting and drying a droplet of water-containing shaped-selected Pt nanocrystals on the gold film (electrochemically active Pt surface area ca. 10 cm^2^), the Si prism was installed in the thin-layer cell by pressing its flat side via an O-ring spacer against the flow cell body. Particular attention was paid to the cleanness of the overall procedure in order to achieve similar experimental conditions as in the beaker cell measurements (see section ‘ATR-FTIRS characterization of structurally well defined Pt nanocrystals’).

#### DEMS setup and measurements

For online product detection, the second compartment of the flow cell was connected via a porous Teflon membrane to a differentially pumped quadrupole mass spectrometer from Pfeiffer Vacuum (QMS 422). A more detailed description of the DEMS setup is given in [58]. The determination of the K* factor necessary for converting mass spectrometric currents (here: CO_2_ signal) into a Faradaic current (here: the Faradaic current for CO_ad_ oxidation) was described in the text.
